# Unpaved road particulate matter emission rates and vehicle-induced transient plume characteristics†

**DOI:** 10.1039/d4ea00055b

**Published:** 2024-08-19

**Authors:** James Kacer, Ralph Altmaier, David M. Cwiertny, Patrick T. O'Shaughnessy

**Affiliations:** a Department of Occupational and Environmental Health, College of Public Health, University of Iowa USA patrick-oshaughnessy@uiowa.edu; b Department of Civil and Environmental Engineering, College of Engineering, University of Iowa USA; c Director, Center for Health Effects of Environmental Contamination, University of Iowa W195 Chemistry Building Iowa City Iowa 52242 USA

## Abstract

Particulate matter (PM) emitted from unpaved rural roads presents a potential inhalation hazard to people living and working near them. In the absence of site-specific exposure data, plume dispersion modeling can be used to predict ambient particulate concentrations in the vicinity of the unpaved roads. Hourly averaged PM_10_ concentrations were measured near a gravel road using an EPA reference method resulting in a geometric mean of 50 μg m^−3^. With these ambient concentrations, the AERMOD plume dispersion model was used to derive a PM emission factor of 444 g/VKT (grams per vehicle kilometer travelled). This result was lower than the emission factor calculated using the EPA's AP-42 guidance for unpaved roads (795 g/VKT). The transient nature of the plume of PM concentrations due to road traffic was also evaluated using a direct-reading instrument. Vehicle speed and wind speed were found to be significant determinants of PM concentration, average PM concentration, and total PM mass for each plume. Each vehicle produced an average concentration of 4096 μg m^−3^ over the duration of the plume. Therefore, residents near the road are potentially exposed to substantially higher short-term concentrations from individual plumes than would be indicated by hourly averages.

## Abstract 2

Particulate matter (PM) emitted from unpaved rural roads presents a potential inhalation hazard to people living and working near them.

## Graphical abstract

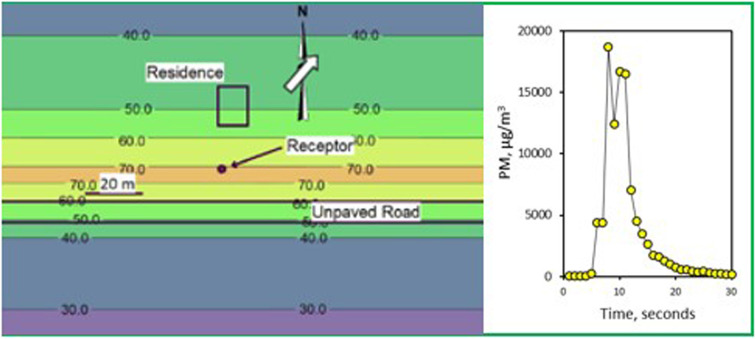

**Environmental significance**

Given their worldwide ubiquity, unpaved roads have the potential to be a large-scale health hazard as a source of exposure to ambient particulate matter (PM) in rural areas. However, their length scale causes PM exposures from unpaved roads to be difficult to assess. This study determined a generalizable PM emission rate using the inverse-modeling approach for application in a plume dispersion model. Notably, 18% of measured concentrations exceeded the 24 hour PM standard. Since the ambient PM is created by passing vehicles, the resulting airborne dust plumes were also investigated. Roadside average concentrations during a plume event were typically 30 times over the PM standard and peak concentrations were over 100 times the standard, therefore stressing the importance of PM from unpaved roads as a potential health hazard.

## Introduction

Ambient particulate matter (PM) has been associated with human health effects such as aggravated asthma, decreased lung function, and other respiratory symptoms.^1,2^ Unpaved roads are common sources of fugitive PM emissions^3,4^ and constitute 32% of all road types in the United States along which are millions of households.^5^ Previous studies have identified potential human health concerns related to inhalation of particulate matter (PM) from unpaved road dust.^6^ The literature review by Khan *et al.*^7^ cited several studies^8–11^ that found associations between road dust PM and various health effects.

Although PM concentrations near unpaved roads can be measured using a number of stationary monitoring methods, their length scale diminishes the usefulness of measurements at any given nearby location to assess community exposure risk. To augment that problem, studies have been conducted using portable, direct reading instruments (DRIs) to measure PM concentrations near unpaved roads. Many of these studies were conducted to compare concentrations measured with DRIs relative to measurements made with federal reference methods (FRMs^12^) or other methods^13–16^ for assessment of risk. For example, Zhao *et al.*^14^ used an aerosol photometer and an optical particle spectrometer alongside several FRMs to compare these DRIs to FRM instruments. The results from the DRIs indicated that the FRMs under-sampled smaller particles based on wind direction. Zhao *et al.*^14^ was the only study found that used DRIs to characterize PM plumes developed from passing vehicles on a time scale less than 1 h. They compared the response from several DRIs operating at 1–5 s sample rates to plumes generated by passing vehicles but did not further characterize the individual plumes relative to, for example, vehicle speed or peak PM concentrations generated.

To supplement and enhance PM exposure information from monitors, plume dispersion modeling has been used to estimate PM concentrations parallel and perpendicular to unpaved roads. The plume dispersion model, AERMOD, has been used for this purpose in several studies involving paved and unpaved roads.^17,18^ AERMOD is the EPA's preferred model for near-field (up to 50 km from the source) analyses of pollutant concentrations^19^ developed for use with steady-state emission sources^20,21^ and is capable of evaluating several types of sources including roads. Askariyeh *et al.*^17^ compared modeled and tracer concentrations of SF_6_ using volume sources to represent roads in the model and found that AERMOD more accurately predicted tracer concentrations at low elevations (0.5 m) than at high elevations (3.5 m and 9.5 m) under low wind speed conditions. Huertas *et al.*^18^ used AERMOD to aid in developing a function to predict PM concentrations emanating perpendicular to the edge of unpaved roads.

In addition to local meteorological information, a primary dispersion model input that influences modeled concentrations is the emission rate of the source. The term “emission rate” is defined for use in AERMOD as the mass per second (units of grams per second) emitted from the source, whereas an “emission factor” for unpaved roads is defined in the EPA AP-42 emission estimation method^22^ as mass per vehicle distance travelled. Studies have been conducted to measure emission rates directly with the use of DRIs.^13,16,23,24^ Kavouras *et al.*^13^ used a vehicle-mounted photometer to estimate PM_10_ emission factors and found statistically significant spatial and temporal variability in emission factors, based on differing road surfaces and vehicle traffic characteristics between sample sites. Edvardsson *et al.*^16^ used aerosol photometers mounted downwind on vehicles to estimate dust emissions and to estimate PM decay with distance from roads to which different dust suppressants had been applied. They found that PM concentrations were dependent on the vehicle speed and type of vehicle. Gillies *et al.*^24^ also used an aerosol photometer to evaluate the differences in emissions from unpaved roads based on vehicle types. Fitz *et al.*^23^ used several DRI types mounted on a trailer to measure vehicle emissions on paved roads and found a close comparison between their results and those derived from the AP-42 methods for paved roads. However, a similar study comparing either measured or estimated emission rates of PM from unpaved roads to estimates using the AP-42 method could not be found in the literature.

Inverse dispersion modeling is an approach that has also been used to estimate particulate emissions from unpaved roads and to identify factors that influence emission factors and airborne particulate concentrations.^25^ When applying inverse modeling, the emission rate is adjusted in the model so that the model output for a receptor location matches a field measurement at that location. Meteorological conditions and other model inputs are typically those observed during field activities for a given sampling duration. For example, the Industrial Source Complex model^19^ was used to estimate the particulate emission rates from unpaved roads in Riverside County, California^26^ using the line source option. Another study used tracers and the line source model to back-calculate emission factors for an unpaved road with low traffic (average 5 vehicles per hour) that reported emission factors ranging from 75 to 298 g/VKT.^27^

Despite the millions of miles of unpaved roads in the United States, there have been relatively few studies conducted to monitor PM concentrations near them. Furthermore, no studies could be found that compared PM emissions from unpaved roads to the AP-42 method for calculating those emissions, nor has a study been performed that systematically characterized the intermittent plumes of PM generated by passing vehicles. Therefore, the primary aim of this study was to determine a fugitive PM_10_ emission rate for rural unpaved roads located in the Midwest United States using the inverse-modeling approach. A sub-aim was to compare the average emission factor estimated by inverse modeling with the emission factor estimated using EPA AP-42 methodology.^22^ A second sub-aim was to evaluate the nature and magnitude of transient PM concentrations developed as short-term plumes by individual moving vehicles.

## Methods

### Study sites

Four sample sites in a rural area of Muscatine County, located in eastern Iowa, were chosen. Sample Site 1 (Fig. S1†) was approximately 15 meters north of the centerline of an unpaved county road oriented east–west. Traffic flow on this road was estimated to be 100 vehicles per day.^28^ The second site, Site 2, (Fig. S1†) was approximately 160 meters south of the same road and was designated as the background site. A third site, Site 3, (Fig. S2†) was used for plume characterization using a direct-reading instrument. This site was on the north side of the road and was located approximately 785 m east of the first site. The fourth site, Site 4, (Fig. S3†) was located on the south side of an unpaved road several miles north of the other three sites and was used for direct-reading plume characterization only when the wind was from a northerly direction.

Road rock in this area was crushed calcite and/or dolomite.^29^ The crushed rock on these unpaved roads was supplemented with electric arc furnace (EAF) slag from a nearby steel foundry. There was no significant difference in PM_10_ concentrations measured near roads supplemented with slag compared to those without supplemental slag when corrected for traffic volume.^29^ Therefore, the four sample sites were considered representative of rural gravel roads in the Midwest. None of the locations had been treated to suppress dust emissions from the road surface prior to sampling.

### Field instrumentation

#### Particulate matter sampling

A beta attenuation monitor (BAM, Model 1020 Continuous Particulate Monitor, Met One Instruments, Inc., Grants Pass, OR), an EPA FRM,^12^ was placed in each of two temporary shelters at Sites 1 and 2 (Fig. S4 and S5†). The BAMs were both configured for PM_10_ analyses on an hourly basis. The intakes for both BAMs were set at approximately 4 m above the ground surface. The terrain near both sites was very flat with the ground elevation at the two sites and the road surface all listed as 198 m above sea level.^30^ The BAMs were operated during warm, dry weather days between July and September 2021, and at least two days after a rain event so that conditions represented those that would create the highest PM emission rates for roads in the area.

Wind direction and velocity data for the sampling period were obtained from a meteorological station (Vantage Vue, Davis Instruments, Hayward, California) located on a neighboring property. Other meteorological data required for the model, such as ceiling height and visibility, were obtained for the National Weather Service facility in Moline, Illinois located 63 km east of Site 1.

Two trail cameras (Model H45, Apeman, Shenzhen, China) were mounted on a post alongside the road at a height of approximately 1 m to obtain traffic counts. The photographs were time- and date-stamped so that traffic counts could be obtained for each one-hour sampling period.

#### Plume characterization

A direct-reading aerosol photometer (Model pDR-1500 Active Personal Particulate Monitor Thermo Fisher Scientific™, Franklin, MA) with a 1 s sample interval was used to measure the rise and fall of particulate concentrations in the plume created as a vehicle passed the sample site. The aerosol photometer measured total particulates with an optimal response relative to particle size between 0.1 and 10 μm.^31^ The aerosol photometer allows for direct collection of PM mass on a filter after it travels through the light-scattering view volume of the instrument. Therefore, the photometer concentration readings were adjusted by calculating a calibration factor that is the ratio of the average of photometer readings over the filter-sample period and the filter gravimetric concentration.

At both locations (Sites 3 and 4), the photometer was positioned approximately 1.5 m above ground level on a tripod and approximately 15 m from the centerline of the adjacent road. Measurements were taken under a variety of wind conditions. A handheld anemometer (Model BTMETER BT-876, Zhuhai, Guangdong, China) and a windsock located near the photometer were used to measure wind velocity and direction during sampling events. Photometer measurements were collected when a project team member drove past the sample location at known speeds of 53, 80, and 113 km h^−1^ (33, 50, and 70 mph) in a mid-sized sedan.

### Dispersion modeling

A commercial software interface package (AERMOD View, version 10.2.1, Lakes Software, Waterloo, Ontario, Canada) containing the latest version of AERMOD^19^ was used to complete modeling runs. This software was used to both estimate PM10 emission rates by inverse modeling and to model the dispersion of PM10 emitted from unpaved roads. Surface meteorological data from a local weather station and upper air data from the National Weather Service were entered into AERMET Version 21112,^32^ a data preprocessor that estimates planetary boundary layer turbulence and formats the data for entry into AERMOD. The AERSURFACE tool^32^ was used to estimate surface characteristics needed for AERMET.

The line volume source option for haul roads was chosen to represent the unpaved road near Site 1. In effect, the line volume source type is a series of adjacent volume sources with a total length equivalent to the road length. This source option was chosen because it includes a meander algorithm which accounts for changes in concentrations due to non-diffusing eddies, and because it better approximated monitoring data in a limited number of studies.^33^ However, ambient concentrations cannot be calculated within the volume source exclusion zone, which is defined in AERMOD as the effective radius of the source (2.15 times the lateral plume dimension plus one meter).^34^ Therefore, the Site 1 sample location (15 m north of the centerline of the road) was placed outside of the exclusion zone (9 m from the centerline of the road).

Each modeling run consisted of a one-hour period corresponding to a one-hour period of BAM data collection. Although road dust emissions are not steady-state at the study site, they were assumed to be constant during the one-hour sampling period of the BAMs at Sites 1 and 2. A receptor height of 4 m was used in the model since that was the height of the air intake of the BAMs. Other model assumptions included a vehicle height of 2 m, a vehicle width of 2 m, and a single-lane road. Applying the AERMOD default factors to the vehicle height and width, the release height was set at 1.7 m with a plume height of 3.4 m and a sigma *z* (initial vertical dilution of the emissions) of 1.58 m; the plume width was set at 8.0 m with a sigma *y* (initial lateral dilution of the emissions) of 3.72 m.^35^

### Emission rates and emission factors

PM10 emission rates are defined in the line volume source option of AERMOD as the total mass of emissions per unit time per source. For the line volume source, the entire line defined in the model is considered a single source, therefore the mass of emissions per unit time was divided by the length of the source to determine mass per time per unit length (g s^−1^ m^−1^). Initially, to determine the emission rate by inverse modeling, hourly background PM_10_ concentrations, *C*_b_, measured at Site 2 were subtracted from the Site-1 PM_10_ concentrations, *C*, measured during the same hours. Since modeled concentration, *C*_m_, is directly proportional to the emission rate, *Q*, inverse modeling to determine an emission rate is only a two-step process. First, an arbitrary emission rate, *Q*_1_, is applied to the model, which is run to obtain a modeled concentration, *C*_m1_, at a receptor location equivalent to the sampling location. The emission rate, *Q*_2_, that will produce a modeled concentration equivalent to the background-compensated sampled concentration is then determined by applying the following concentration ratio:^36^1
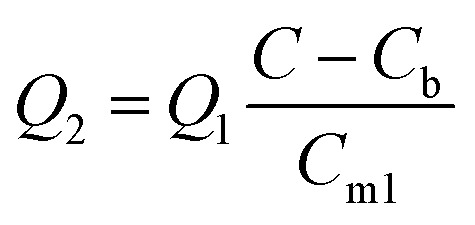


The geometric mean (GM) of the emission rates determined by inverse modeling in units of g s^−1^ m^−1^ was divided by the number of vehicles (V) traveling on the unpaved road during a sampled hour (g s^−1^ m^−1^ V^−1^) to determine an emission factor in units of mass per vehicle kilometer traveled (g/VKT) and converted to mass per vehicle mile traveled (lb/VMT) for comparison with the AP-42 method. The EPA AP-42 equation developed for emissions from light-duty vehicles traveling on publicly accessible unpaved roads is defined as:^22^2
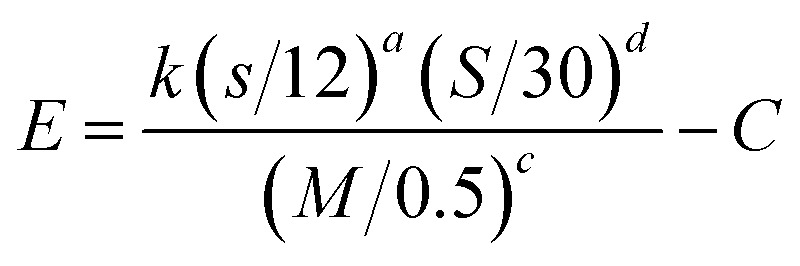
where, *E* is the emission factor (lb/VMT), *s* is the surface material silt content, *M* is the surface material moisture content, *S* is the mean vehicle speed (mph), and *C* is an emission factor for 1980's vehicle fleet exhaust, brake wear and tire wear. The constants have values of *k* = 1.8, *a* = 1, *c* = 0.2, *d* = 0.5, and *C* = 0.00047 lb/VMT for emissions of PM_10_ from public unpaved roads.^22^ For the calculation of *E*, the maximum allowable silt percentage of 16% for Class-A crushed rock was used based on information provided by the local county engineer. Likewise, the county engineer stated that the crushed rock typically had a moisture content of 0.8%. Because the speed of vehicles traveling the road near Site 1 was not measured as part of the field work, the emission factor was calculated assuming an average speed of 80 km h^−1^ (50 mph), which is near the posted speed limit.

There are several relevant differences in these methods for determining an emission factor. The AP-42 method includes ranges for vehicle weights while AERMOD requires vehicle height and width as inputs. The AP-42 method includes site-specific data for silt content and moisture of the unpaved road surface, whereas AERMOD ignores these road characteristics. Furthermore, the AP-42 method directly accounts for vehicle speed while inverse modeling only implicitly accounts for vehicle speed as it contributes to the resulting emission factor.

#### Sensitivity analyses

Sensitivity analyses were conducted on both the AP-42 emission calculations and the inverse modeling approach. The sensitivity of the AP-42 emission factor to variations of silt content percentages, silt moisture content percentages, and vehicle speeds were calculated using a range of values for these variables that are representative of the expected conditions at the sample site.

Vehicle characteristics have been found to have a significant effect on the calculation of emission factors.^37^ Therefore, the sensitivity of the inverse-modeled per-vehicle emission rate to variations in vehicle height and width were also calculated using a range of values for these variables that are representative of the expected conditions at the sample site. Changes were made to vehicle height and width while holding the meteorological conditions applied to the model constant and representative of a warm, summer day near noon.

A sensitivity analysis was also undertaken to investigate the relative effect of different wind speeds and directions on the value of the inverse-modeled emission rate. A single hour of meteorological data was applied to AERMOD representing a typical summer afternoon and the concentration at the receptor (Site 1 sample location) was set at 50 μg m^−3^. The source descriptors applied were the same as those described in “Dispersion Modeling.” Inverse modeling was applied to determine the emission rate while varying wind direction between 180° to 270° by every 22.5°, and wind speed was set at 1.6, 8, 12, and 16 km h^−1^ (1, 5, 7.5, 10 mph).

### Model simulations of roadside concentrations

To simulate roadside concentrations under varying wind speeds and directions, AERMOD was run at two wind speeds, 1.6 km h^−1^ (1 mph) and 16 km h^−1^ (10 mph), and three wind directions, 180°, 225°, and 270°, while holding all other modeling inputs constant. The emission rate applied during all simulations was chosen to simulate typical measured concentrations.

### Transient concentration measurement analysis

An individual plume measured with the aerosol photometer was defined as the fugitive PM generated by the passage of one car from the time the background concentration was exceeded until concentrations returned to background levels. Plume PM characteristics determined from the recorded series of photometer measurements included maximum concentration (μg m^−3^); average concentration (μg m^−3^); time from background concentration to maximum concentration (seconds); plume decay residence time (seconds); and total mass measured per plume (μg). After an initial increase in concentration, the plume was assumed to follow first-order decay. The decay rate was therefore determined from the slope of the linear regression of log-concentration relative to time. Not all plumes decayed in this manner, therefore, a criterion was established such that an *R*^2^ > 0.6 of the linear regression was required to calculate the plume residence time. The total mass measured per plume was calculated by back-solving the mass of PM sampled by the photometer knowing the average concentration of a plume, the span of time of the plume and the sample flow rate of the photometer. The total mass therefore provides an indication of both the longevity and magnitude of a plume relative to other plumes.

Multiple regression analysis was applied to determine whether these plume characteristics were significantly affected by the continuous independent variables of vehicle speed, wind speed, and wind direction. Minitab® version 21.2 (Minitab, LLC, State College, PA) was used for all statistical analyses. Significance was tested at the *α* = 0.05 level.

## Results and discussion

### Hourly PM_10_ measurements and emission rates

The BAMs at Sites 1 and 2 recorded 871 hours and 558 hours of PM_10_ concentrations, respectively. These hourly results were highly skewed (Fig. S6†), therefore, the GM (and geometric standard deviation, GSD) were computed to indicate the central tendency and variation of the concentrations. A GM of 50 (3.3) μg m^−3^ and 26 (2.0) μg m^−3^ were obtained at Site 1 and Site 2, respectively. Additional descriptive statistics are provided in Table S1† of the supplement.

During the entire sampling campaign, there were 34 24 h periods starting and ending at midnight for which 24 h averages could be calculated. These daily averages had a GM of 77 μg m^−3^. The EPA NAAQS PM_10_ 24 h limit of 150 μg m^−3^ is not to be exceeded more than once per year. However, 6 (18%) of the 24 h averages were over the limit with a maximum of 330 μg m^−3^.

There were 365 hours during which the BAMs at Sites 1 and 2 were both operational during the same hours to enable background correction of the Site 1 measurements. However, those used for inverse modelling were limited to hours when the wind was from 180° to 270°; that is, when a south-to-southwest wind was blowing from the road toward the Site 1 sampler. PM measurements when south-easterly winds were blowing from 90° to 180° were not used because a north-south unpaved road to the southeast of the sampler at Site 2 could have contributed to particulate concentrations above background levels (Fig. S1†). Hours with no measurable wind were also not used because AERMOD will not provide results for a wind speed of zero.^31,32^ A total of 64 paired BAM concentration measurements from Sites 1 and 2 met the wind-related selection criteria and were used for the inverse-modeling process. The GM for the emission rate and related emission factor determined by inverse modeling are listed in Table 1. Additional descriptive statistics are provided in Table S2.†

**Table 1:** Geometric means of emission rates and resulting emission factor

Emission rate (g s^−1^)	Emission rate/vehicle (g s^−1^ V^−1^)	Emission factor (g/VKT)
1.09	0.26	444

The GM emission factor predicted by inverse modeling, 444 g/VKT, is approximately half the emission factor calculated using AP-42 methodology, 795 g/VKT. The GSD of the modeled emission factor (4.33) was high, indicating a large amount of variability in inverse-modeled emission rates and indicates the potential for much higher actual values than the GM value. Some of the variability in emission rates may be explained by the short-term variations in wind speed that can be observed under low wind conditions, which may not be adequately accounted for in the AERMOD simulations.^38^ Many of the observations in this study were recorded under relatively low wind conditions. Furthermore, a prior study estimating the particulate emission rates from paved and unpaved roads by inverse modeling^26^ found little correlation between the emission factors determined using the model and the emission factors estimated using AP-42 methodology across several study sites, which may have been associated with the high variability found in the current study. O'Shaughnessy and Altmaier^36^ also obtained an inverse-modeled emission rate for hydrogen sulfide emanating from swine facilities that was lower than those determined from direct emission measurement studies such as the study by Cowherd *et al.*^38^ that was used as the basis of the AP-42 method applied in this study. Given a choice between the different emission rates obtained using the two methods, O'Shaughnessy and Altmaier^36^ suggest that the emission rate developed from inverse modeling using AERMOD may be the most accurate rate to apply when also using AERMOD to model the dispersion of the same contaminant under similar meteorological and terrain conditions.

### Sensitivity analyses

Results of the sensitivity analysis of variables affecting the value of the AP-42 emission factor are given in Table S3.† The emission factor is most sensitive to changes in the road material moisture content, decreasing 113-fold per 1% increase in moisture content. The amount of silt increased the emission 50-fold per 1% increase in silt content. However, there is a much greater range of possible silt content that results in the greatest difference between lowest and highest emission factor values. These results highlight the importance of obtaining accurate measurements of the silt content and moisture content of the material used on unpaved road surfaces when estimating emission factors for unpaved roads. Changes in vehicle speed also had a relatively large effect of 162 g/VKT across the range analyzed of 48–113 km h^−1^ (30–70 mph).

Table S4† summarizes the sensitivity of the inverse-modeled emission rate to variations in vehicle heights and widths representative of the type of vehicles expected on roads where PM_10_ samples were collected. Whereas vehicle widths ranging from 1.73 m to 5.03 m had no discernible effect on the resulting emission rate, changes in vehicle height from 1.42 m to 3.81 m increased the emission rate 1.3-fold. The model is therefore more sensitive to changes in vehicle height than changes in width.

Results for the sensitivity analysis of the effects of wind direction and wind speed on the inverse-modeled emission rate are given in Fig. S7.† The emission rate was not sensitive to changes in the wind direction at wind speeds of 1.6 km h^−1^ and 8 km h^−1^, which may be explained by the initiation of plume meander when wind speeds are less than 2 m s^−1^ (7.2 km h^−1^).^38^ At 12 km h^−1^ and 16 km h^−1^, the emission rate changes with wind direction between 180° to 270°, with a minimum observed at approximately 250° and a maximum at 270°.

### Simulated roadside concentrations

**Fig. 1:**
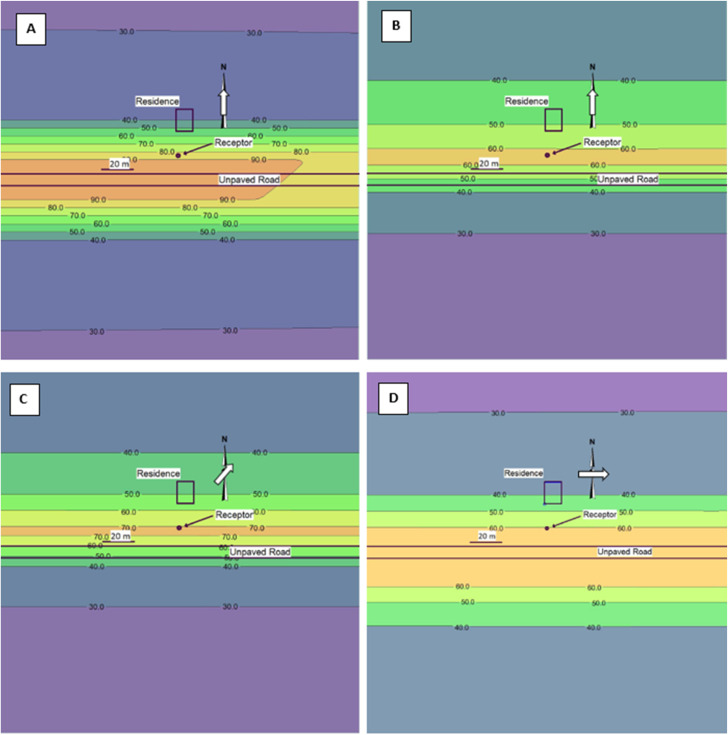
Modeled PM_10_ plumes at different wind directions and wind speeds using an emission rate of 1.3 g s^−1^ and added background concentration of 26 μg m^−3^. The same distance scale is used for all plots (note 20 m scale bar). Plots A and B result from applying a 180° wind at 1.6 km h^−1^ (1 mph) (A) and 16 km h^−1^ (10 mph) (B). Plots C and D result from applying a 225° wind (C) and a 270° wind (D), both with wind speeds at 16 km h^−1^ (10 mph).

The GM emission rate per vehicle given in Table 1 (0.26 g s^−1^ V^−1^) was applied to run AERMOD while assuming 5 vehicles per hour to achieve an emission rate of 1.3 g s^−1^ and also adding a background concentration of 26 μg m^−3^ to produce plots showing modeled concentrations typical of measured concentrations. A receptor height of 1.5 m above ground level was used because it was within the approximate breathing zone of an adult human. Modeling results are shown in Fig. 1. The concentration contour plots developed for all wind directions at 1.6 km h^−1^ were nearly identical, therefore, only the plot for the results when modelling a 180° wind direction are given (Fig. 1A), which indicates that modeled concentrations are not sensitive to wind direction at low wind speeds, a result supported by the sensitivity analysis shown in Fig. S7.†

For wind blowing at 16 km h^−1^ directly from the south (Fig. 1B) and southwest (Fig. 1C), PM_10_ concentrations were higher downwind of the source, as expected, with the concentrations decaying faster at the higher wind speed, also as expected. For wind blowing directly from the west and, therefore, parallel to the road (Fig. 1D), the concentration profiles are nearly symmetrical and centered on the volume line source representing the gravel road. This scenario represents limited dispersion of the PM as the plume follows the axis of the road rather than dispersing predominantly to one side of the road. Of the three wind directions, the highest concentration at the receptor is produced at 225°. In effect, this wind angle moves the high concentrations developed by a nearly parallel wind towards one side of the road, in this case, towards the receptor. Furthermore, the inverse-modeled emission rate for a given measured concentration is lowest near 225° as shown in Fig. S7.† This result implies that, when the same emission rate is applied to the model, the spatial concentrations obtained with a 225° wind are higher than for any other wind angle as shown in Fig. 1.

### Vehicle-generated PM_10_ plumes

Measurements using the aerosol photometer were conducted over 15 non-contiguous days that were each at least 3 days after a rain event. Each pass of a car past the photometer was defined as a plume. A total of 147 plumes were recorded and represented a variety of conditions based on varying wind speeds and wind directions. A time-series plot of vehicle-generated plumes from one sampling episode is given in Fig. S8.† The GMs of the five evaluated plume characteristics are provided in Table 2. Additional descriptive statistics are summarized in Table S5.†

**Table 2:** Geometric means of vehicle-generated plume characteristics

Average conc. (μg m^−3^)	Max conc. (μg m^−3^)	Total mass (μg)	Time to peak (s)	Residence time (s)
4278	18743	3.4	8.2	7.4

**Fig. 2:**
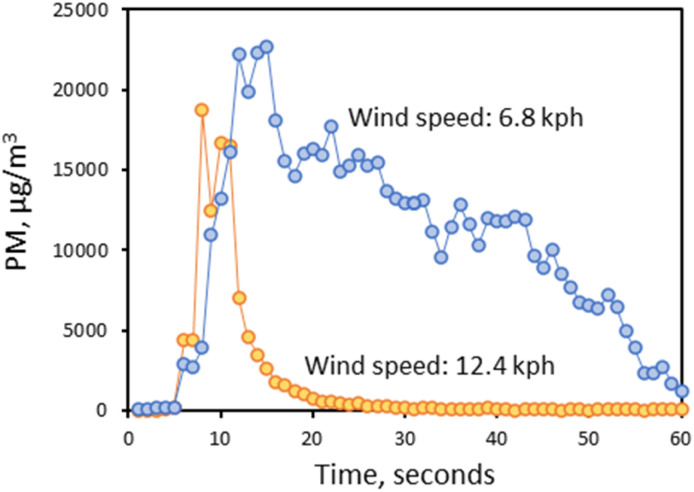
Comparison of PM plume profiles for two different wind speeds, with approximately the same wind directions, using a direct-reading instrument with one-second logging.

**Fig. 3:**
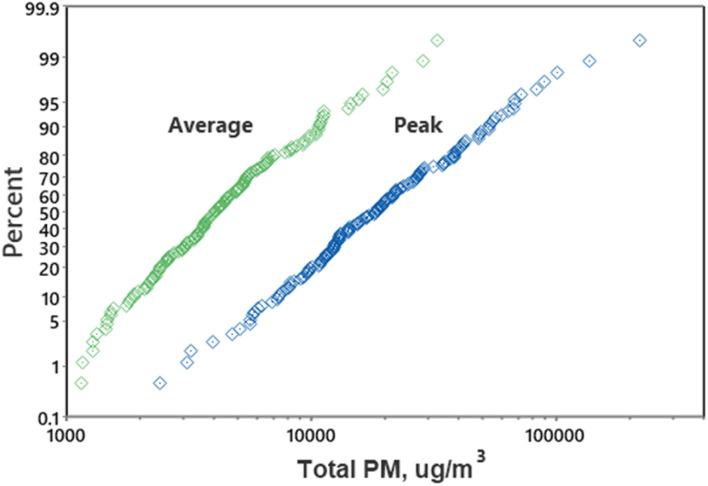
Probability plots of aerosol photometer average and peak PM concentrations for each plume.

Fig. 2 provides a comparison of the maximum (peak) concentrations and the plume residence duration for two plumes with similar vehicle speeds and wind directions but different wind speeds. The two plots in Fig. 2 demonstrate that the plume generally had a longer decay residence time at low wind speeds, as expected, which results in a longer PM exposure period. Fig. 3 provides probability plots of the average plume concentrations and corresponding peak concentrations, which demonstrate that peak concentrations were typically 4- to 5-fold higher than average concentrations.

The wind speed and direction varied somewhat during trial periods with each trial generally lasting 20 to 35 minutes. The mean of the differences between the beginning and ending wind speeds for each trial was 2.3 km h^−1^, and the mean of the differences between the beginning and ending wind directions for each trial (sample day) was 46.1°.

#### Regression analysis

Results of the multiple regression analyses of the sample runs are summarized in Table S6.† In an initial statistical analysis, vehicle moving direction was found to be non-significant (*p* < 0.05), so it was excluded from further analyses.

Vehicle speed was found to be a significant predictor of both maximum and average PM concentration and the total mass, but not for the time-related variables of time to the peak concentration and plume-decay residence time. This suggests that as a vehicle passes the sampler, the time to the maximum concentration and plume residence time will stay relatively constant under constant meteorological conditions with different vehicle speeds, while the average concentration and total PM mass will increase with vehicle speed, therefore, the PM emission rate was also enhanced by vehicle speed. Wind speed was a significant predictor for all dependent variables. However, wind direction was significant only for dust plume residence time, consistent with the finding in our previous research^29^ This finding suggests the intuitive explanation that the long plume of airborne dust generated by a passing car will be pushed down the road when the wind is parallel to it, thus extending its decay residence time relative to a plume that is pushed laterally by a wind perpendicular to the road.

### Limitations

This study design did not include an evaluation of the effects of vehicle size, speed, and weight on hourly PM_10_ measurements measured at Site 1 with the BAM. Likewise, vehicles were not identified and quantified by type during hourly and daily sampling.

## Conclusions

Hourly concentrations of PM_10_ measured near an unpaved, gravel road in eastern Iowa during dry, warm conditions resulted in a GM of 50 μg m^−3^. More importantly 6 of 34 24 h average concentrations exceeded the PM_10_ NAAQS limit. The emission factor calculated by inverse modeling using hourly PM_10_ field measurements was approximately half the emission factor calculated using the AP-42 methodology. Given the number of variables and the differences between the two methodologies, this represents a relatively close agreement, although the geometric standard deviation for the modeled emission factor of 4.33 was high, thus indicating a very large range in estimated emission rates.

Results obtained when monitoring the plume generated by a passing vehicle indicated that wind speed and vehicle speed had significant effects on maximum PM and average concentrations, and the PM total mass collected. The direct reading aerosol monitor results demonstrated that the peak concentration of PM_10_ resulting from the passage of a single vehicle can be over 10 000 μg m^−3^. A short-term concentration level of that magnitude could result in adverse health effects for sensitive individuals.

## Data availability

The data that support the findings of this study are available from the corresponding author, PTO, upon reasonable request at: patrick-oshaughnessy@uiowa.edu.

## Author contributions

J. K.: conceptualization, methodology, investigation, formal analysis, data curation, writing – original draft; P. T. O.: conceptualization, methodology, project administration, funding acquisition, resources, software, supervision, writing – review & editing; R. A.: investigation, writing – review & editing; D. M. C.: conceptualization, funding acquisition, project administration, resources, writing – review & editing.

## Conflicts of interest

There are no conflicts to declare.

## Supplementary Material

EA-004-D4EA00055B-s001
