# Supplementary material for: Unpaved road particulate matter emission rates and vehicle-induced transient plume characteristics
Source: Environ Sci Atmos. 2024 Aug 19;4(9):1042–50. doi: 10.1039/d4ea00055b (PMC11342916; doi:10.1039/d4ea00055b)
Supplement: EA-004-D4EA00055B-s001 [file EA-004-D4EA00055B-s001.pdf]

Supplementary Information (SI) for Environmental Science: Atmospheres.
This journal is © The Royal Society of Chemistry 2024

Supplemental Information

Use of Inverse Modeling to determine Particulate Emission Rates from Rural Unpaved Roads

James Kacer[a], Ralph Altmaier[b], David M. Cwiertny[c,d] Patrick T. O'Shaughnessy[b]

[a] Center for Health Effects of Environmental Contamination, W195 Chemistry Building, University of Iowa, Iowa City, Iowa, 52242

[b] Department of Occupational and Environmental Health, College of Public Health, University of Iowa

[c] Department of Civil and Environmental Engineering, College of Engineering, University of Iowa

[d] Director, Center for Health Effects of Environmental Contamination, W195 Chemistry Building, University of Iowa, Iowa City, Iowa, 52242

Tables

Table S1 Descriptive Statistics of Hourly PM_{10} Measurements at Sites 1 and 2.

Parameter	Site 1 μg/m^3	Site 2 μg/m^3
Sample Size	871	558
Geometric Mean	50 (3.3[1])	26 (2.0)
Arithmetic Mean	106 (162[2])	34 (30.6)
Minimum	1	0
Maximum	1717	256

[1]Geometric standard deviation
[2]Standard deviation

Table S2 Descriptive Statistics of Emission Rates and Emission Factors (n = 64).

Parameter	Emission Rate, g/s	Emission Rate/ Vehicle, g/s-V	Emission Factor, g/VKT
Geometric Mean	1.09 (4.9[1])	0.26 (4.3[1])	444 (4.3[1])
Arithmetic Mean	3.34 (5.67[2])	0.67 (1.06)	1153 (1826)
Minimum	0.02	0.01	8.5
Maximum	25.70	4.28	8430

[1]Geometric standard deviation
[2]Standard deviation

Table S3. Summary of Sensitivity Analysis of Variables Affecting the Value of the AP-42 Emission Factor

Variable	Variable Range	Variable Mid-value	Unit Change[1]	Range Change[2]
% Silt	1 - 16	8	50	745
% Moisture	0.4 – 1.2	0.8	-113	-90
Speed, kph	48 - 113	80	2.5	162

[1]Change in emission factor per unit change of the variable
[2]Change in emission factor from lowest to highest value of the variable

Table S4. Sensitivity of the Inversely Modeled Emission Rate (G/S) to Changes in Vehicle Width and Vehicle Height

		Vehicle Width, m					
		1.73	1.83	2.21	2.59	2.59	5.03
Vehicle height, m	1.42	0.647[1]					0.650
	1.45	0.648	0.649[2]				0.651
	2.03	0.671		0.672[3]			0.676
	3.53	0.798			0.798[4]		0.801
	4.11	0.857				0.857[5]	0.860
	3.81	0.825					0.829[6]

[1]Small car

[2]Medium car

[3]Pickup truck/SUV

[4]Grain truck

[5]Tractor-trailer

[6]Farm combine, transport width and height

Table S5 Descriptive Statistics for Vehicle-Generated Plume Characteristics (n = 147).

	Average Conc. μg/m^3	Max Conc. μg/m^3	Total Mass μg	Time to Peak sec	Residence Time sec
Geomean	4278 (1.96[1])	18743 (2.19[1])	3.4 (2.57[1])	8.2 (1.98[1])	7.4 (5.2[1])
Minimum	1137	2385	0.3	2.0	2.2
Maximum	32780	221017	54.0	62.0	147

[1]Geometric standard deviation

Table S6. Regression Coefficients for Plume Sampling Outcomes

	ln Maximum Concentration	ln Average Concentration	ln Time to Peak Concentration	ln Residence Time	ln Total Mass
vehicle speed	0.021***	0.016***	0.001	0.003	0.022***
wind speed	-0.047*	-0.048**	-0.108***	-0.056***	-0.132***
wind direction	0.006	0.004	0.004	-0.007*	0.001

p: *<0.05, **<0.01, ***<0.001

Figures

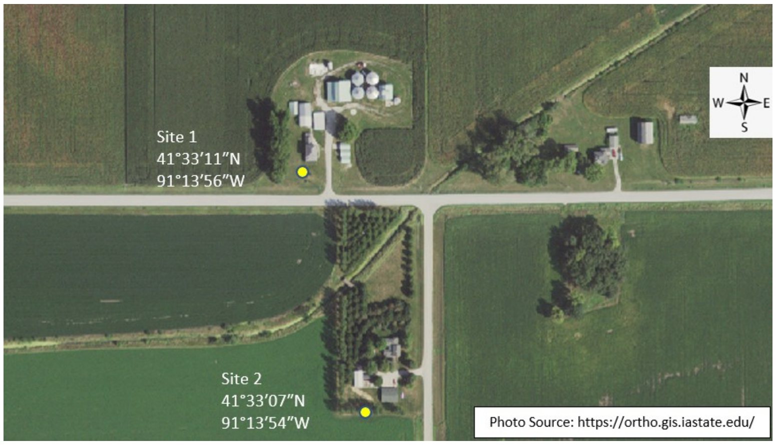

Fig. S1. Site 1 and Site 2 BAM sample locations.

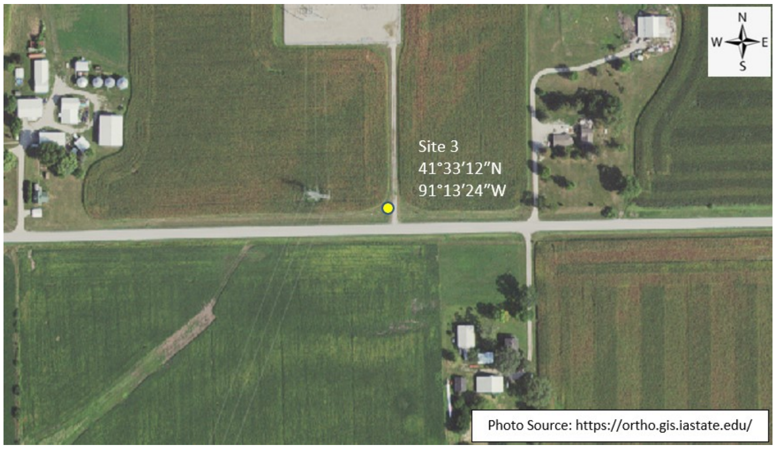

Fig. S2. Site 3 southerly-wind aerosol photometer sampling site.

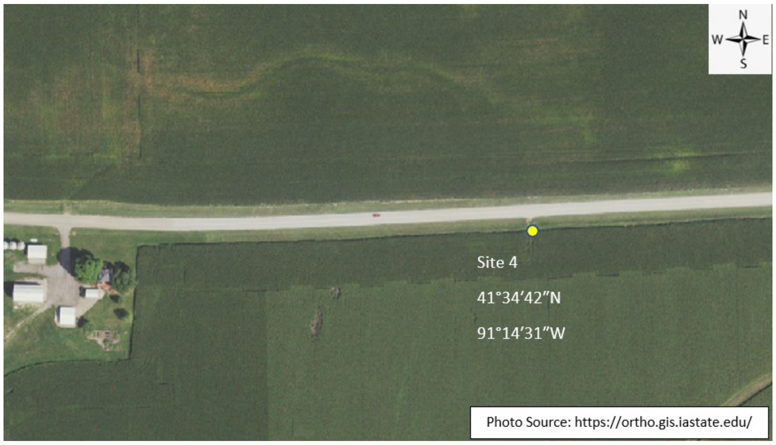

Fig. S3. Site 4 northerly-wind aerosol photometer sampling site.

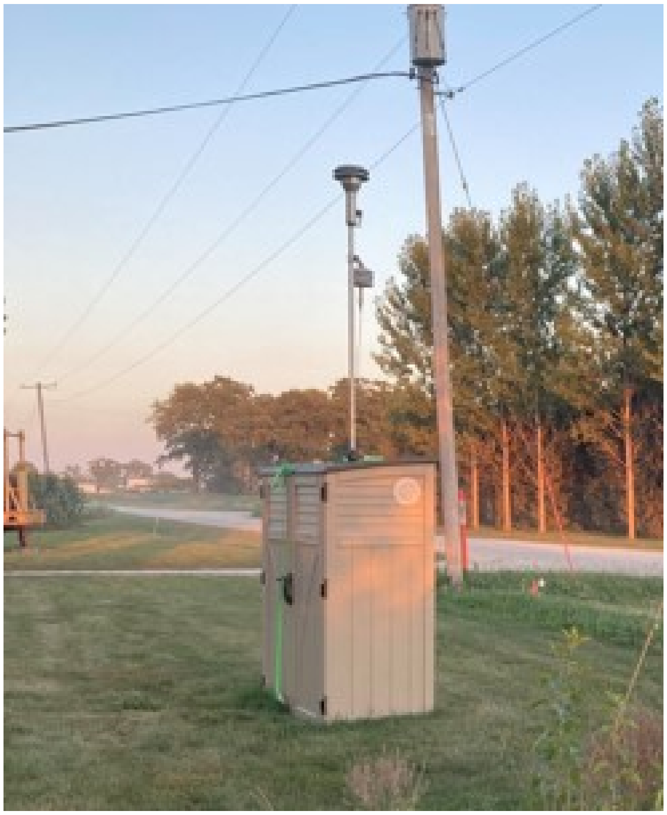

Fig. S4. The Site 1 sample shed containing a BAM monitor positioned near a rural unpaved road.

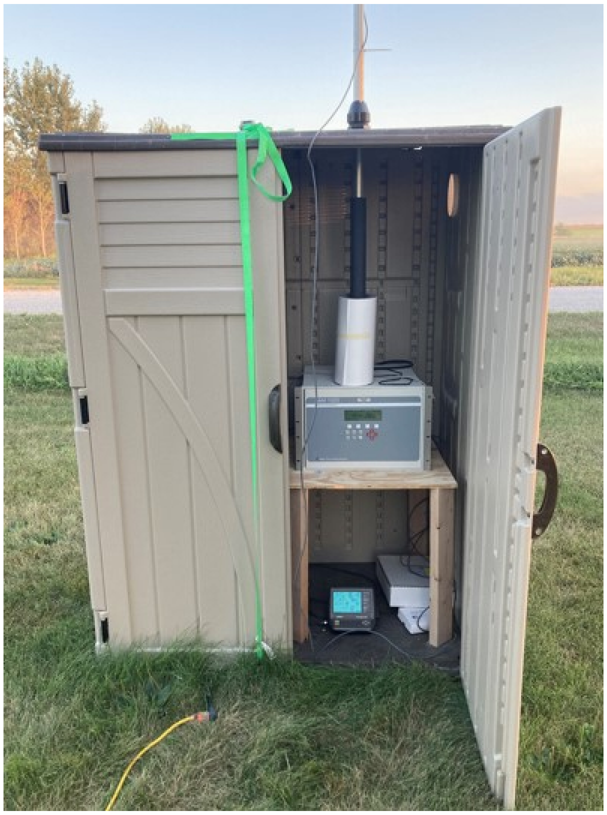

Fig. S5. The Site 1 sample shed showing configuration of the BAM monitor.

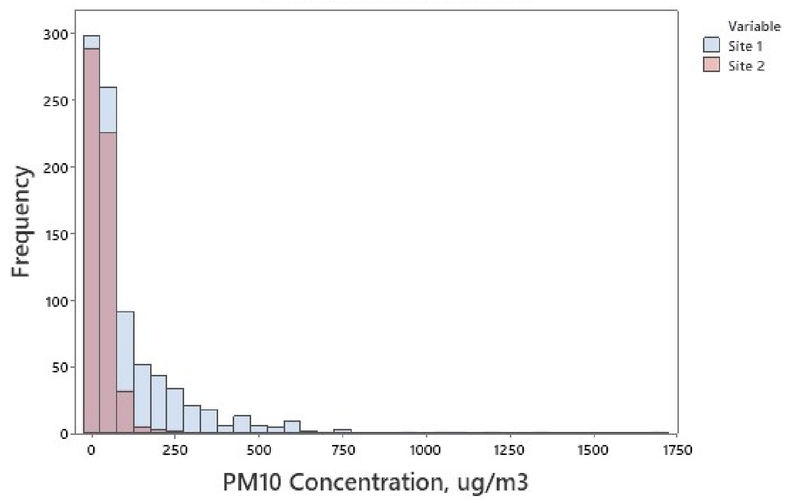

Fig. S6. Site 1 and Site 2 PM_{10} concentration frequency distributions.

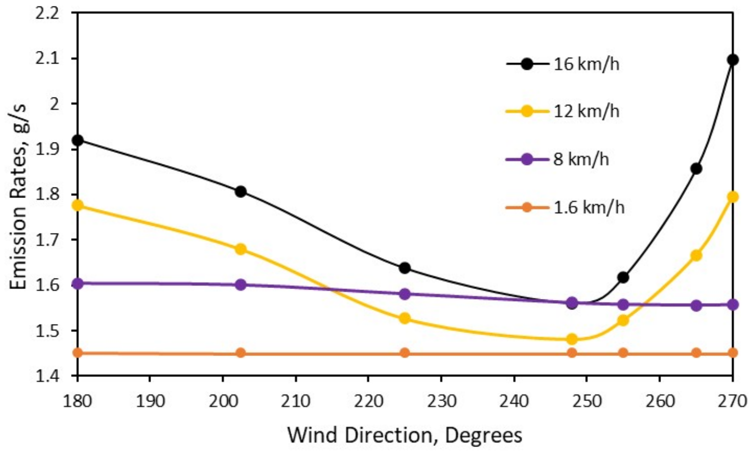

Fig. S7. Inverse modeled emission rates given varying wind directions and wind speeds while all other model inputs remained constant over a one-hour meteorological period.

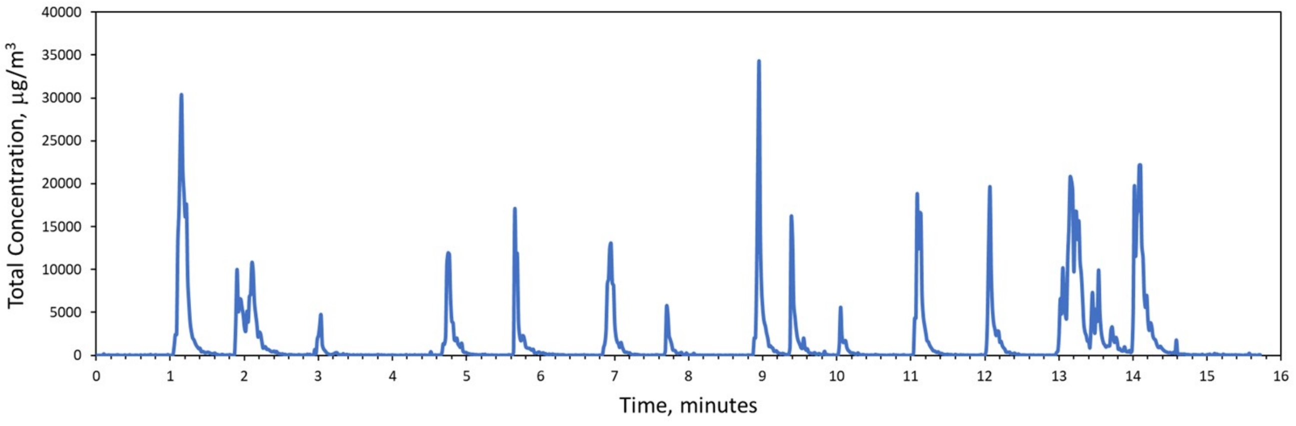

Fig. S8. Particulate matter plume profiles resulting from a car passing the aerosol photometer every one to three minutes.
